# Multimodality management for chronic subdural hematoma in China: protocol and characteristics of an ambidirectional, nationwide, multicenter registry study

**DOI:** 10.1186/s41016-024-00356-5

**Published:** 2024-01-25

**Authors:** Tao Liu, Zhihao Zhao, Jinhao Huang, Xide Zhu, Weiliang Chen, Kun Lin, Yunhu Yu, Zhanying Li, Yibing Fan, Mingqi Liu, Meng Nie, Xuanhui Liu, Chuang Gao, Wei Quan, Yu Qian, Chenrui Wu, Jiangyuan Yuan, Di Wu, Chuanxiang Lv, Shiying Dong, Liang Mi, Yu Tian, Ye Tian, Jianning Zhang, Rongcai Jiang

**Affiliations:** 1 Department of Neurosurgery, Tianjin Medical University General Hospital, Tianjin, China; 2 Ministry of Education, Tianjin Neurological Institute, Key Laboratory of Post Neuro-injury Neuro-repair and Regeneration in Central Nervous System, Tianjin Medical University General Hospital, Tianjin, China; 3 Department of Neurosurgery, Linyi People’s Hospital, Shandong, China; 4 Department of Neurosurgery, Haining People’s Hospital, Zhejiang, China; 5 Department of Neurosurgery, Fujian Provincial Hospital, Fujian, China; 6 Department of Clinical Research Center for Neurological Disease, the People’s Hospital of HongHuaGang District of ZunYi, Guizhou, China; 7 Department of Neurosurgery, Kailuan General Hospital, Hebei, China; 8 Department of Neurosurgery, Tianjin First Central Hospital, Tianjin, China; 9 Department of Neurosurgery, The First Hospital of Jilin University, Changchun, China

**Keywords:** Chronic subdural hematoma, Multimodality therapy, Outcome, Recurrence

## Abstract

**Background:**

Despite its prevalence, there is ongoing debate regarding the optimal management strategy for chronic subdural hematoma (CSDH), reflecting the variability in clinical presentation and treatment outcomes. This ambidirectional, nationwide, multicenter registry study aims to assess the efficacy and safety of multimodality treatment approaches for CSDH in the Chinese population.

**Methods/design:**

A multicenter cohort of CSDH patients from 59 participating hospitals in mainland China was enrolled in this study. The treatment modalities encompassed a range of options and baseline demographics, clinical characteristics, radiographic findings, and surgical techniques were documented. Clinical outcomes, including hematoma resolution, recurrence rates, neurological status, and complications, were assessed at regular intervals during treatment, 3 months, 6 months, 1 year, and 2 years follow-up.

**Result:**

Between March 2022 and August 2023, a comprehensive cohort comprising 2173 individuals who met the criterion was assembled across 59 participating clinical sites. Of those patients, 81.1% were male, exhibiting an average age of 70.12 ± 14.53 years. A historical record of trauma was documented in 48.0% of cases, while headache constituted the predominant clinical presentation in 58.1% of patients. The foremost surgical modality employed was the burr hole (61.3%), with conservative management accounting for 25.6% of cases. Notably, a favorable clinical prognosis was observed in 88.9% of CSDH patients at 3 months, and the recurrence rate was found to be 2.4%.

**Conclusion:**

This registry study provides critical insights into the multimodality treatment of CSDH in China, offering a foundation for advancing clinical practices, optimizing patient management, and ultimately, improving the quality of life for individuals suffering from this challenging neurosurgical condition.

**Trial registration:**

ChiCTR2200057179

## Backgroud

Chronic subdural hematoma (CSDH) represents a prevalent neurological condition linked to a spectrum of risk factors, encompassing advanced age, cranial trauma, anticoagulant therapy, and underlying medical comorbidities [1]. Recently, there has been a global upswing in the incidence of CSDH, notably among elderly individuals afflicted with concurrent disease [2, 3]. This escalating incidence, concomitant with heightened recurrence rates and resultant disability attributed to CSDH, has cast a substantial burden upon healthcare systems and has merited augmented attention from the medical community [4, 5].

The therapeutic paradigm for CSDH has undergone refinement over the years, with surgical intervention emerging as the primary modality. Procedures such as burr hole, twist drill, and craniotomy are enlisted to effectuate hematoma evacuation and mitigate intracranial pressure [4, 6]. Nonetheless, consensus regarding the optimal surgical approach remains elusive, influenced, in part, by individual experience and preferences of surgeons. In recent years, there has been a burgeoning interest in multimodality treatment strategies for CSDH that combine surgical intervention with postoperative drainage, hematoma irrigation, and pharmacological therapy [7, 8]. These multimodal therapeutic approaches are conceived not only to address the exigent clearance of acute hematomas but also to target the underlying pathophysiological processes that contribute to CSDH recurrence, encompassing inflammatory cascades, neovascularization, and coagulopathies [3]. Despite the escalating adoption of these comprehensive CSDH treatments, a conspicuous lacuna persists in the availability of extensive real-world data to inform clinical decision-making and facilitate the development of clinical guidelines.

This multicenter registry study of the CSDH (MRCSDH) trial endeavors to ensure the encompassment of a diverse patient cohort and the meticulous acquisition of comprehensive data. The principal objectives of the MRCSDH trial encompass as follows: (1) to elucidate the clinical characteristics of Chinese patients with CSDH and assess the efficacy of various treatment modalities in reducing CSDH recurrence rates; (2) to evaluate the safety of these treatments and identify potential complications; and (3) to undertake an exhaustive exploration of patient-specific variables associated with favorable outcomes such as age, comorbidities, medication history, and hematoma characteristics. In this report, we systematically present the rationale, methodology, and implementation of this study, along with its strengths and potential limitations.

## Methods/design

### Overview

The MRCSDH is an ongoing, multicenter, ambidirectional, non-interventional trial funded by the National Natural Science Foundation and the Zhao Yicheng Medical Science Foundation of Tianjin. A committee, composed of members from Tianjin Medical University General Hospital and several neurosurgery centers, was responsible for the trial's design and organization. Prior to the formal study, a preliminary investigation was conducted to determine center eligibility. Research centers failing to meet the following criteria were excluded: (1) hospitals lacking certification or licensing; (2) hospitals without appropriate facilities and equipment; (3) those lacking the requisite expertise and experience; and (4) hospitals not in compliance with ethical requirements.

### Study objective

The primary objective of this investigation is to gain insight into the characteristics of CSDH patients in China, alongside an exploration of the prevailing therapeutic modalities. Concurrently, the study aims to appraise the efficacy of diverse treatment methodologies in mitigating the recurrence rates. In terms of safety, the study will evaluate the safety of these treatments and identify potential complications through follow-up assessments. Furthermore, we will utilize the real-world research data acquired to investigate patient-specific factors that may influence treatment outcomes, including age, comorbidities, medication history, and hematoma characteristics.

The MRCSDH trial represents a significant advancement in neurosurgery by examining the efficacy and safety of comprehensive treatment strategies for CSDH in the Chinese population. The results of this study have the potential to enhance clinical practice guidelines, optimize treatment protocols, and elevate the standards of care afforded to individuals grappling with the intricate challenges posed by this neurological disorder.

### Patient selection and screening

All patients diagnosed with CSDH at participating hospitals will be eligible candidates for this study, with an anticipated enrollment of 3,000 CSDH patients from March 2022, to February 2024. Inclusion criteria: (1) individuals diagnosed with CSDH, regardless of gender; (2) supratentorial, unilateral, or bilateral CSDH on CT scans (in cases of diagnostic uncertainty, MRI imaging may be performed); (3) patients with complete clinical and imaging data; and (4) individuals who provide informed consent. Exclusion criteria are as follows: (1) patients lacking critical baseline demographic, clinical, and imaging data; (2) individuals unable to complete the trial due to poor compliance; and (3) patients in critical condition nearing the end of life.

### Baseline data collection

During data collection, we employed a digital service platform system (https://login.medbit.cn). After completing training courses, each research center proceeded with real-name registration. Following registration, new case records were created for participants who met the inclusion criteria, and data uploads and follow-up monitoring were conducted according to the visit schedule. The completeness, consistency, and reliability of data for each patient were verified by clinical research coordinators at the local centers. The entire data collection process adhered to the Helsinki Declaration (2013 revision) and was supervised and guided by the Ethics Committee of Tianjin Medical University General Hospital.

Our case report form includes the following information: (1) patient demographic data, including name, gender, age, height, weight, ethnicity, occupation, date of hospital visit, initial consultation location (emergency room, outpatient clinic, external referral, inpatient ward). (2) Clinical presentation, including medical history, personal history, medication history, cause of onset (presence of trauma history), time from trauma to diagnosis, severity of trauma (mild, moderate, severe), primary and secondary symptoms at onset, initial mRS score at the first visit. (3) Radiological characteristics and laboratory test at admission, length of hospital stay, costs incurred, other concomitant symptoms and diseases during hospitalization, mRS scores at discharge, 3 months, 6 months, 12 months, and 24 months. (4) Treatment modalities: Conservative treatment (various medications), surgical treatment (burr hole drainage, craniotomy, embolization treatment, and so on), or multimodality treatment.

All data collection was conducted by clinical frontline personnel with a medical background, and any disputes were resolved by clinical research associates.

### Clinical management

The study incorporates various treatment modalities, including simply observed, conservative treatment, burr hole, craniotomy, endoscopy, embolization, multimodality treatment, and others. The choice of treatment may vary depending on individual patient differences. The ultimate treatment decision should be made through discussion and agreement between the physician and the patient. However, we have also established the following standardized treatment options for reference: observation (simply observed): for some small and asymptomatic CSDH cases, physicians may opt for observation only, monitoring the progression of the hematoma. This approach is often suitable for elderly patients. Conservative treatment: conservative treatment typically involves bed rest, hypertension control, discontinuation or reduction of anticoagulant medication use, and symptomatic management (such as pain control). This method is suitable for some patients, especially those who refuse surgery or have a higher surgical risk. Burr hole: burr hole surgery is a classic method for treating CSDH. It involves small-hole surgery to evacuate the hematoma through these openings, and a drainage tube may be left in place to facilitate fluid drainage. Craniotomy: in some cases, craniotomy may be necessary, especially when the hematoma is large, closely adherent to brain tissue, or requires more extensive surgical intervention. Endoscopy: endoscopic surgery can also be used for CSDH treatment. It involves small-hole surgery and the introduction of an endoscope to directly visualize and evacuate the hematoma. Embolization: in certain situations, especially when CSDH is associated with vascular abnormalities, embolization may be an option. It involves blocking abnormal blood vessels through interventional procedures to reduce bleeding and the occurrence of CSDH. Multimodal treatment: multimodal treatment combines various therapeutic approaches to better meet the needs of patients and specific disease conditions. This may include surgery, pharmacological treatment, and other therapeutic measures, generally reserved for refractory CSDH.

All patients will undergo routine laboratory tests and standardized care. Physicians and family members will be responsible for monitoring the progression of patients' neurological symptoms. When their symptoms significantly improve, they may be discharged and followed up according to a predetermined schedule.

### Follow-up assessment

We have established follow-up time points at 3 months, 6 months, 12 months, and 24 months after treatment, and follow-up responsibilities will be assigned to clinical research coordinators at each center. Before discharge, patients will be informed to return for scheduled reassessment promptly. In the event that a patient does not return for follow-up, investigators will make attempts to contact the patient or their family through telephone or other available means to explain the situation and complete the outcome assessment. If all follow-up attempts prove unsuccessful, further contact will be discontinued, and the patient will be recorded as lost to follow-up. Any occurrences of death, both inside and outside the hospital, should be documented, and possible causes investigated.

### Outcomes measurements

The primary outcome is the neurological functional status (mRS score) at 6 months after treatment. Secondary outcomes include changes in hematoma volume, occurrence of adverse events, mortality rate, hospitalization costs, and recurrence rate within 2 years. Adverse events encompass all complications that occur during the treatment period, including newly diagnosed diabetes, recent psychiatric diagnoses, infections, seizures, acute cardiovascular events, and others. Hematoma absorption volume refers to the difference between the hematoma volume calculated through imaging examinations (such as CT) after treatment and the baseline hematoma volume at admission. The mortality rate represents the all-cause mortality rate during the treatment period.

### Statistical analysis

Categorical variables are presented as percentages, while continuous variables are expressed as mean ± standard deviation (SD) or median with interquartile range (IQR). In univariate analysis, comparisons of continuous variables were conducted using the *t*-test or Mann-Whitney *U* test, and comparisons of categorical variables were performed using the *χ*^2^ test or Fisher’s exact test when applicable. In cohort studies, propensity score matching (PSM) was employed to adjust for confounding factors. A *p* value of < 0.05 (two-tailed) was considered statistically significant. Statistical analysis in this article was performed using SPSS (version 25.0, IBM, New York, USA), and baseline demographic and clinical characteristics of included and excluded patients, as well as initiating and collaborating units, were compared.

## Results

**Fig. 1:**
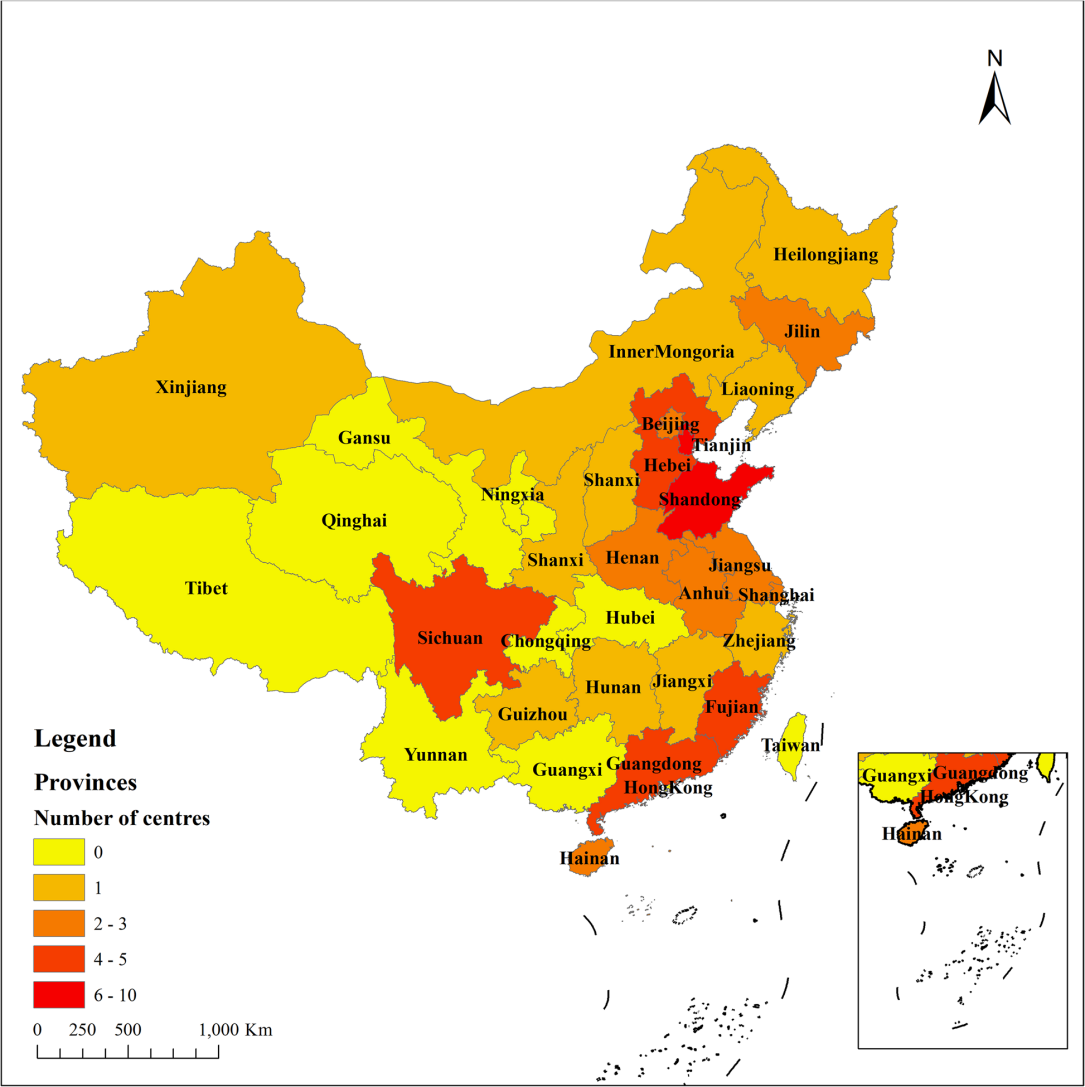
The geographical locations of participating centers in the MRCSDH trial

As patient recruitment and follow-up are still ongoing, we are currently summarizing the period from Mar 2022 to August 2023. After 3 months of continuous recruitment, a total of 59 hospitals were ultimately identified. These hospitals declared their reliable research capabilities and willingness to commit to this study. Table 1 presents the names of participating centers, and Fig. 1 displays the geographical locations of all hospitals involved in the research.

**Table 1:** Medical centers in China participating in the study

Number	Medical centers
1	Tianjin Medical University General Hospital
2	People’s Hospital of Honghuagang District, Zunyi
3	The Fourth Affiliated Hospital of China Medical University
4	Rudong County People's Hospital
5	Shanxi Provincial People’s Hospital
6	Zhujiang Hospital of Southern Medical University
7	Baoding First Central Hospital
8	West China Hospital of Sichuan University
9	The First Affiliated Hospital of Shandong First Medical University (Shandong Provincial Qianfoshan Hospital)
10	Fuyang Hospital of Anhui Medical University
11	The First People’s Hospital of Changde
12	The First Affiliated Hospital of Xiamen University
13	Jiangsu Province Hospital
14	Tianjin Hospital of Integrated Traditional Chinese and Western Medicine
15	The Affiliated Hospital of Southwest Medical University
16	Tangshan People’s Hospital
17	The First Affiliated Hospital of Xinjiang Medical University
18	Suining Central Hospital
19	The 900^th^ Hospital of Joint Logistic Support Force of PLA
20	Linyi People’s Hospital
21	The First Hospital of Jilin University
22	Tianjin Fifth Central Hospital
23	Haikou People’s Hospital
24	Ordos Central Hospital
25	The First Affiliated Hospital of Wannan Medical College
26	Tianjin People’s Hospital
27	Nanjing Drum Tower Hospital
28	Shanghai East Hospital
29	Santai County People’s Hospital
30	Lingcheng District People's Hospital, Dezhou
31	Tianjin First Central Hospital
32	Heze Municipal Hospital
33	The First Affiliated Hospital of Xinxiang Medical University
34	Langfang People's Hospital
35	Anyang People’s Hospital
36	Fujian Provincial Hospital South Branch
37	Cangzhou Central Hospital
38	Tianjin Huanhu Hospital
39	Haining People’s Hospital
40	Kailuan General Hospital
41	Binzhou Medical University Hospital
42	Xi’an Central Hospital
43	South China Hospital Affiliated to Shenzhen University
44	Tangshan Gongren Hospital
45	Hianan General Hospital
46	The First Affiliated Hospital of Harbin Medical University
47	Shanghai Sixth People’s Hospital
48	Siping Central People’s Hospital
49	Anfu County People’s Hospital
50	Beijin Tiantan Hospital
51	Peking Union Medical College Hospital
52	Jieyang People’s Hospital
53	The Second Hospital of Shandong University
54	The Third People’s Hospital of Henan Province
55	The First Affiliated Hospital of Fujian Medical University
56	Jian’ou Municipal Hospital
57	Liaocheng Brain Hospital
58	Liaocheng Second People’s Hospital
59	Huoshan County Hospital

**Fig. 2:**
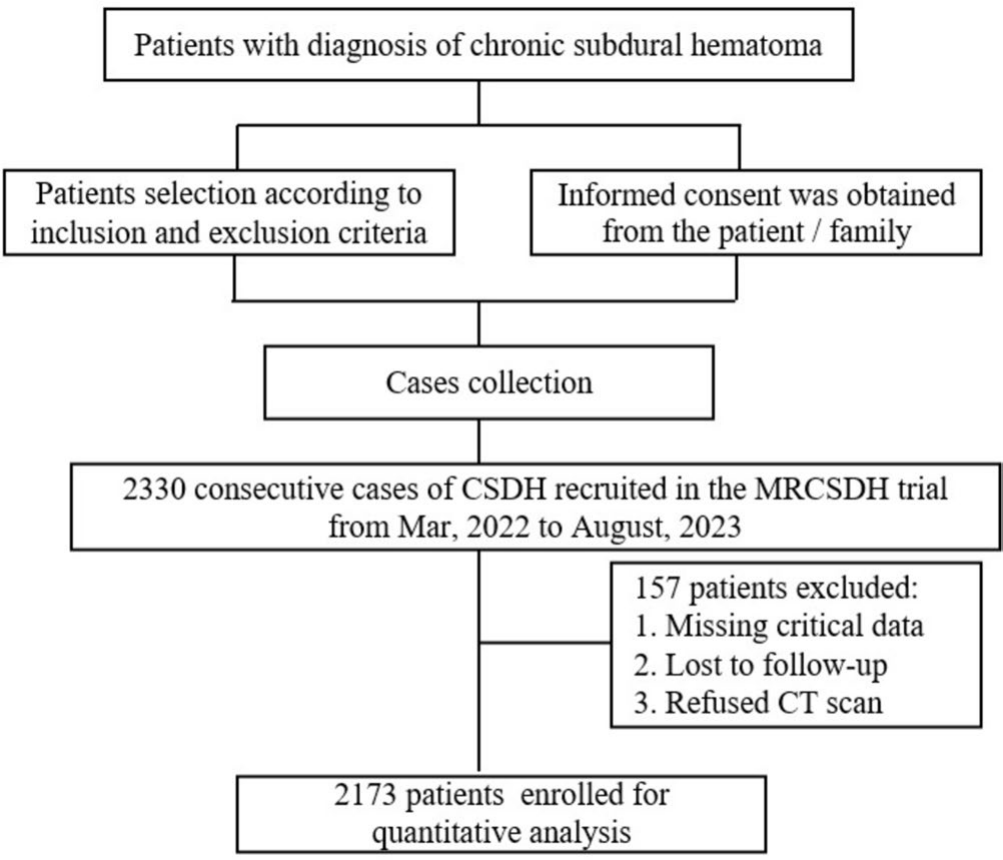
Flow chart to illustrate the design of the MRCSDH trial

Out of the 2330 patients initially included in the study, 157 were excluded following rigorous screening, resulting in a final cohort of 2173 patients who met the inclusion criteria. The detailed patient enrollment process is illustrated in Fig. 2. Visits and assessments will be conducted in accordance with the schedule (Table 2). In addition to the history of trauma, level of the hospital and residence classification, the included patients had similar baseline characteristics as those excluded (Table 3).

**Table 2:** Visit and assessment schedule in the MRCSDH trial

Activities	Screening or baseline	Pre-treatment	At discharge	3 M ± 7D	6 M ± 7D	12M ± 7D	24 ± 7D
Informed consent	✓						
Inclusion/exclusion criteria	✓						
General information	✓						
Medical history	✓						
Medication history	✓						
Personal history	✓						
Physical examination		✓	✓				
Vital sign		✓	✓	✓	✓	✓	✓
Neurological symptoms		✓	✓	✓	✓	✓	✓
Hematoma volume by CT/MRI	✓	✓	✓	✓	✓	✓	✓
Lab test		✓	✓	✓	✓	✓	✓
Pregnancy test		✓					
Electrocardiogram		✓					
Treatment strategy		✓	✓				
Current medication	✓	✓	✓	✓	✓	✓	✓
mRS assessment		✓	✓	✓	✓	✓	✓
GCS		✓	✓	✓	✓	✓	✓
Adverse events			✓	✓	✓	✓	✓
Recurrence			✓	✓	✓	✓	✓

*CT* computed tomography, *MRI* magnetic resonance imaging, *mRS* modified Rankin scale, *GCS* Glasgow coma scale

**Table 3:** Baseline characteristics of included and excluded in the MRCSDH trial

Parameters	Total (*N* = 2330)	Included (*N* = 2173)	Excluded (157)	*P* value
Age, years	70.08 ± 14.70	70.12 ± 14.53	69.01 ± 18.175	0.564
Male	1891(81.2)	1762(81.1)	129(82.2)	0.738
Nationalities				0.629
Han	2239 (96.1)	2087(96.0)	152(96.8)	
Other	91 (3.9)	86 (4.0)	5 (3.2)	
Initial assessment location				0.454
Emergency department	1165 (50.0)	1094 (50.3)	71 (45.2)	
Outpatient clinic	1015 (43.6)	943 (43.4)	72 (45.9)	
External referral	61 (2.6)	55 (2.5)	6 (3.8)	
Inpatient ward	89 (3.8)	81 (3.7)	8 (5.1)	
Level of the hospital				
Tertiary A	1964 (84.3)	1841 (84.7)	123 (78.3)	0.034
History of trauma	1163 (49.9)	1044 (48.0)	119 (75.8)	< 0.001
Body height	167.55 ± 10.40	167.62 ± 10.11	165.07 ± 17.43	0.327
Weight	65.58 ± 11.40	65.61 ± 11.32	64.60 ± 14.23	0.639
Occupation				0.579
Peasantry	870 (37.3)	804 (37.0)	66 (42.0)	
Worker	152 (6.5)	143 (6.6)	9 (5.7)	
Retiree	569 (24.4)	536 (24.7)	33 (21.0)	
Other	739 (31.7)	690 (31.8)	49 (31.2)	
Residence classification				< 0.001
First-tier	84 (3.6)	72 (3.3)	12 (7.6)	
Second-tier	1216 (52.2)	1101 (50.7)	115 (73.2)	
Other	1030 (44.2)	1000 (46.0)	30 (19.1)	
Admission mRS, median [IQR]^a^	2 [1–3]	2 [1–3]	1 [1–3]	0.229

^a^84 patients in the excluded cohort missing admission mRS data

Table 4 presents baseline demographics and characteristics of included patients between initiating organizations and collaborating organizations. Among them, 81.1% were male, with an average age of 70.12. Regarding the type of treatment, there were significant differences between the initiating organization and collaborating organizations. The initiating organization tended to favor conservative treatment, while the collaborating organizations leaned towards burr hole. The difference in admission mRS between the two groups has statistical significance. Overall, the patients included by the initiating organization had more severe symptoms compared to those from collaborating organizations, but there was no statistically significant difference in favorable outcome and recurrence at 3 months.

**Table 4:** Baseline demographic and Characteristics between initiating organization and collaborating organizations

Parameters	Total (*N* = 2173)	Initiating organization cohort (*N* = 527)	Collaborating organizations cohort (*N* = 1646)	*P* value
Age, years	70.12 ± 14.53	70.08 ± 16.18	70.14 ± 13.95	0.938
Male	1762 (81.1)	405 (76.9)	1357 (82.4)	0.004
Nationalities				0.023
Han	2087 (96.0)	515 (97.7)	1572 (95.5)	
Other	86 (4.0)	12 (2.3)	74 (4.5)	
Main manifestation				
Headache	1262 (58.1)	298 (56.5)	964 (58.6)	0.413
Dizziness	877 (40.4)	205 (38.9)	672 (40.8)	0.433
Limbs powerless	1121 (51.6)	265 (50.3)	856 (52.0)	0.492
Nausea	110 (5.1)	33 (6.3)	77 (4.7)	0.149
Type of treatment				
Conservation	557 (25.6)	299 (56.7)	258 (15.7)	< 0.001
Burr hole	1333 (61.3)	133 (25.2)	1200 (72.9)	< 0.001
History of trauma	1044 (48.0)	172 (32.6)	872 (53.0)	< 0.001
Body height	167.62 ± 10.11	168.10 ± 11.25	167.43 ± 9.61	0.238
Weight, median	65.61 ± 11.32	67.30 ± 12.39	65.00 ± 10.84	< 0.001
Occupation				< 0.001
Peasantry	804 (37.0)	12 (2.3)	792 (48.1)	
Worker	143 (6.6)	23 (4.4)	120 (7.3)	
Retiree	536 (24.7)	201 (38.1)	335 (20.4)	
Other	690 (31.8)	291 (55.2)	399 (24.2)	
Patients completed 6 months follow-up	2117 (97.4)	523 (99.2)	1594 (96.8)	0.002
Admission mRS, Median [IQR]	2 [1–3]	3 [2–4]	2 [1–3]	< 0.001
Favorable outcome at 3 months	1931 (88.9)	478 (90.7)	1453 (88.3)	0.123
Recurrence at 3 months	52 (2.4)	13 (2.5)	39 (2.4)	0.899

## Discussion

The MRCSDH is the first multicenter, nationwide CSDH study conducted in mainland China. To ensure diversity within the patient population and comprehensive data collection, this study spans across most regions of mainland China. Our research is dedicated to exploring the characteristics of CSDH patients and the existing treatment modalities, assessing the effectiveness of various comprehensive treatment methods in reducing CSDH recurrence rates, and evaluating the safety of these treatments while identifying potential complications. Additionally, we aim to investigate the factors influencing individualized treatment strategies, such as age, comorbidities, medication history, and hematoma characteristics. Based on these findings, we aspire to enhance the implementation of optimized treatment approaches.

CSDH was first reported by Wepfer in 1657 as ‘delayed stroke’ [5], and since Hulke's report of the first case treated with burr hole in 1883, numerous methods have been employed for the treatment of CSDH [9]. Literature on CSDH varies significantly in reporting mortality rates (0–32%), but the primary concern is recurrence rates, ranging from 0 to 76%, and approximately 10%-20% of recurrent cases necessitate repeat surgery [10, 11]. Rauhala et al. found that the average cost per patient with no recurrence was 3820 euros (median, 3370 euros), whereas the average cost per recurrent patient was 8850 euros (median, 7110 euros) [4]. Therefore, reducing recurrence is crucial for mitigating complications and costs. However, there are multiple factors influencing the prognosis of CSDH, including age, hematoma volume, midline shift, anticoagulant medication use, and drainage duration, among others [1]. Exploring CSDH personalized treatment strategies for recurrence and long-term neurological prognosis based on the characteristics of the Chinese population is necessary.

A search of PubMed yielded 13 randomized controlled trials related to CSDH, including two from mainland China. The first study, titled “Effect of ATorvastatin On Chronic subdural Hematoma (ATOCH),” commenced in January 2014 [12]. Prior research had found that atorvastatin could mobilize endothelial progenitor cells (EPCs) to promote angiogenesis while also inhibiting vascular endothelial growth factor, reducing inflammation [13, 14]. Based on these hypotheses and the team's clinical experience, the ATOCH team considered atorvastatin as a potential non-surgical treatment strategy for CSDH. To our excitement, the ATOCH team’s findings were published in 2018, garnering attention from scholars both domestically and internationally [15]. The study included 196 patients with CSDH from 31 medical centers. The research revealed that atorvastatin could facilitate the hematoma absorption of CSDH, reducing the likelihood of transitioning to surgical treatment due to hematoma enlargement and worsening symptoms during conservative treatment. These research findings are poised to change the current situation of lacking effective non-surgical treatments for CSDH, enabling many elderly CSDH patients to avoid the risks of surgery, reduce treatment costs, and benefit a larger patient population. The second study was a phase II clinical trial [16]. It found that atorvastatin combined with low-dose dexamethasone could expedite the absorption of CSDH hematomas and improve neurological function. These results suggested a potential synergistic effect between the two drugs in immunoregulation and vascular repair. However, given the limited sample size, this proof-of-concept clinical trial lacks credibility. Nevertheless, given these promising results, trials evaluating the synergistic effect of these two drugs in CSDH treatment are currently underway [17].

Furthermore, there are some noteworthy international studies related to CSDH. Santarius et al. found that the use of a drain after burr-hole drainage in CSDH patients was associated with a reduced recurrence and mortality at 6 months. Additionally, irrigation at body temperature during burr hole surgery for CSDH can reduce the risk of recurrence by over half [18]. Hutchinson et al. found that, compared to a placebo, dexamethasone did not significantly improve outcomes in CSDH patients, but a more superior performance in preventing hematoma recurrence [19]. The latest research in 2023 has concluded this debate, with evidence from Miah demonstrating the ineffectiveness of dexamethasone for CSDH [20].

While these results are disappointing, efforts to improve the prognosis of CSDH patients continue. The number of patients and participating centers in most CSDH studies conducted in China currently falls short of expectations, and confounding factors affecting treatment outcomes make the effectiveness and safety of various treatment methods elusive, especially for refractory CSDH patients. We look forward to the MRCSDH trial resolving the current predicament, as it is a nationwide registry that can provide an ample sample for prognostic analysis of each treatment strategy, assess the safety of these treatments and identify potential complications, and support the development of prognostic prediction models.

The MRCSDH trial has several advantages. Firstly, considering the characteristics of Chinese patients, this trial contributes to providing more applicable treatment recommendations and guidelines for the Chinese region. The trial encompasses multiple medical centers, ensuring diversity within the patient population and comprehensive data collection, which enhances the external validity and representativeness of the research. By utilizing real-world data, it reflects multimodal treatments in actual clinical practice, helping to understand the real-world effectiveness of treatments beyond idealized conditions in a research setting. The diverse objectives of the MRCSDH trial, including evaluating treatment effectiveness, safety, and patient-specific factors, contribute to providing more comprehensive clinical information.

However, the MRCSDH trial also has some limitations. Since it is an observational study without random allocation, there may be baseline differences between the treatment and control groups, making it challenging to establish causality. Data quality and consistency might be issues, including data missing, incompleteness, and reporting errors, which could potentially affect the reliability of the study. Research on multimodality treatments provides diverse data but may also lead to result complexity, making it difficult to draw definitive conclusions.

## Conclusion

The MRCSDH trial offers the advantages of extensive data collection, an ambidirectional design, and real-world data, but it is essential to be mindful of data quality issues and potential selection bias. This study contributes to enhancing our understanding of the treatment of CSDH, but caution is needed when interpreting and applying its results, particularly considering the complexity of treatment diversity and patient-specific factors.

## Data Availability

The datasets used and/or analyzed during the current study are available from the corresponding author on reasonable request.

## Abbreviations

CSDH: Chronic subdural hematoma
MRCSDH: Multicenter Registry Study of CSDH
SD: Standard deviation
IQR: Interquartile range
PSM: Propensity score matching
ATOCH: ATorvastatin On Chronic subdural Hematoma
EPCs: Endothelial progenitor cells

## References

[1] Zhu F, Wang H, Li W, Han S, Yuan J, Zhang C (2022). Factors correlated with the postoperative recurrence of chronic subdural hematoma: an umbrella study of systematic reviews and meta-analyses. EClinicalMed.

[2] Almenawer SA, Farrokhyar F, Hong C, Alhazzani W, Manoranjan B, Yarascavitch B (2014). Chronic subdural hematoma management: a systematic review and meta-analysis of 34,829 patients. Ann Surg.

[3] Kolias AG, Chari A, Santarius T, Hutchinson PJ (2014). Chronic subdural haematoma: modern management and emerging therapies. Nat Rev Neurol.

[4] Rauhala M, Helén P, Huhtala H, Heikkilä P, Iverson GL, Niskakangas T (2020). Chronic subdural hematoma-incidence, complications, and financial impact. Acta Neurochirurgica.

[5] Uno M (2023). Chronic subdural hematoma-evolution of etiology and surgical treatment. Neurologia Medico-Chirurgica.

[6] Duerinck J, Van Der Veken J, Schuind S, Van Calenbergh F, van Loon J, Du Four S (2022). Randomized trial comparing burr hole craniostomy, minicraniotomy, and twist drill craniostomy for treatment of chronic subdural hematoma. Neurosurgery.

[7] Baschera D, Tosic L, Westermann L, Oberle J, Alfieri A (2018). Treatment standards for chronic subdural hematoma: results from a survey in Austrian, German, and Swiss Neurosurgical Units. World Neurosurgery.

[8] Cenic A, Bhandari M, Reddy K (2005). Management of chronic subdural hematoma: a national survey and literature review. The Canadian journal of neurological sciences Le journal canadien des sciences neurologiques.

[9] Weigel R, Krauss JK, Schmiedek P (2004). Concepts of neurosurgical management of chronic subdural haematoma: historical perspectives. British journal of neurosurgery.

[10] Weigel R, Schmiedek P, Krauss JK (2003). Outcome of contemporary surgery for chronic subdural haematoma: evidence based review. Journal of neurology, neurosurgery, and psychiatry.

[11] Ducruet AF, Grobelny BT, Zacharia BE, Hickman ZL, DeRosa PL, Andersen KN et al. The surgical management of chronic subdural hematoma. Neurosurgical review. 2012;35(2):155-69; discussion 69. doi:10.1007/s10143-011-0349-y. 10.1007/s10143-011-0349-y 21909694

[12] Jiang R, Wang D, Poon WS, Lu YC, Li XG, Zhao SG (2015). Effect of ATorvastatin On Chronic subdural Hematoma (ATOCH): a study protocol for a randomized controlled trial. Trials.

[13] Liu Y, Wei J, Hu S, Hu L (2012). Beneficial effects of statins on endothelial progenitor cells. The American journal of the medical sciences.

[14] Blum A (2014). HMG-CoA reductase inhibitors (statins), inflammation, and endothelial progenitor cells-New mechanistic insights of atherosclerosis. BioFactors (Oxford, England).

[15] Jiang R, Zhao S, Wang R, Feng H, Zhang J, Li X (2018). Safety and efficacy of atorvastatin for chronic subdural hematoma in Chinese patients: a randomized clinical trial. JAMA Neurol.

[16] Wang D, Gao C, Xu X, Chen T, Tian Y, Wei H (2020). Treatment of chronic subdural hematoma with atorvastatin combined with low-dose dexamethasone: phase II randomized proof-of-concept clinical trial. Journal of Neurosurgery.

[17] Jiang RC, Wang D, Zhao SG, Wang RZ, Kang Z, Zhu XG (2021). Atorvastatin combined with dexamethasone in chronic subdural haematoma (ATOCH II): study protocol for a randomized controlled trial. Trials.

[18] Bartley A, Bartek J, Jakola AS, Sundblom J, Fält M, Förander P (2023). Effect of irrigation fluid temperature on recurrence in the evacuation of chronic subdural hematoma: a randomized clinical trial. JAMA Neurol.

[19] Hutchinson PJ, Edlmann E, Bulters D, Zolnourian A, Holton P, Suttner N (2020). Trial of dexamethasone for chronic subdural hematoma. The New England journal of medicine.

[20] Miah IP, Holl DC, Blaauw J, Lingsma HF, den Hertog HM, Jacobs B (2023). Dexamethasone versus surgery for chronic subdural hematoma. The New England journal of medicine.

